# The Treatment of Hiccough

**Published:** 1891-09

**Authors:** 


THE TREATMENT OF HICCOUGH.—The *Gazette Hebdomadaire* gives the following for hiccough. The local treatment is to compress the phrenic nerve and the pneumogastric in the neck by pressure with the index finger, which will often cause cessation of spasms. The medical treatment consists in the administration of a coffee-spoonful of vinegar mixed with a little powdered sugar, or the following formula may be used:

| | | | | |
|---|---|---|---|---|
| R. | Subnitrate of bismuth, | - | | grs. xlv |
| | Oxide of zinc, | | | |
| | Valerianate of zinc, | | | |
| | Powdered calumba, | - | aa | ʒ j |
| | Powdered opium, | - | - | grs. jss |
| | Essence of anise, q. s. | | | |

Mix thoroughly and give half a teaspoonful of this powder in a wineglassful of sweetened water.—*Southern Clinic.*

